# Copy number analysis of the low-copy repeats at the primate *NPHP1* locus by array comparative genomic hybridization

**DOI:** 10.1016/j.gdata.2016.04.008

**Published:** 2016-04-19

**Authors:** Bo Yuan, Pengfei Liu, Jeffrey Rogers, James R. Lupski

**Affiliations:** a Department of Molecular and Human Genetics, Baylor College of Medicine, Houston, TX 77030, USA; b Baylor Miraca Genetics Laboratories, Houston, TX 77030, USA; c Human Genome Sequencing Center, Baylor College of Medicine, Houston, TX 77030, USA; d Department of Pediatrics, Baylor College of Medicine, Houston, TX 77030, USA; e Texas Children's Hospital, Houston, TX 77030, USA

**Keywords:** Array comparative genomic hybridization, *NPHP1*, Primate evolution, Low-copy repeats

## Abstract

Array comparative genomic hybridization (aCGH) has been widely used to detect copy number variants (CNVs) in both research and clinical settings. A customizable aCGH platform may greatly facilitate copy number analyses in genomic regions with higher-order complexity, such as low-copy repeats (LCRs). Here we present the aCGH analyses focusing on the 45 kb LCRs [1] at the *NPHP1* region with diverse copy numbers in humans. Also, the interspecies aCGH analysis comparing human and nonhuman primates revealed dynamic copy number transitions of the human 45 kb LCR orthologues during primate evolution and therefore shed light on the origin of complexity at this locus. The original aCGH data are available at GEO under GSE73962.

**Table:** 

Specifications	
Organism/cell line/tissue	*Papio anubis*, *Macaca mulatta*, *Pongo abelii*, *Gorilla gorilla*, *Pan troglodytes*, *Homo sapiens*
Sex	NA
Sequencer or array type	Agilent customized aCGH with 8x60K format
Data format	Analyzed
Experimental factors	Normal
Experimental features	Copy number estimation of the human 45 kb LCRs or its nonhuman primate orthologues in the test samples using NA10851 as control
Consent	NA
Sample source location	Houston, TX USA

## 1 Direct link to deposited data

http://www.ncbi.nlm.nih.gov/geo/query/acc.cgi?acc=GSE73962.

## 2 Experimental design, materials and methods

### 2.1 Samples

Thirty-two DNA samples were tested for copy number of the 45 kb LCRs in humans or its orthologue in nonhuman primates. These included DNA samples of seven individuals from the International HapMap project (http://hapmap.ncbi.nlm.nih.gov/), three human cell lines, eight patients with homozygous *NPHP1* deletion, one baboon (*Papio Anubis*), two rhesus macaques (*Macaca mulatta*), one orangutan (*Pongo abelii*), three gorillas (*Gorilla gorilla*), and seven chimpanzees (*Pan troglodytes*). The DNA sample of NA10851, a human individual with known copy number of the 45 kb LCRs, was used as the universal control for the intra/inter-species aCGH experiments.

### 2.2 aCGH — design

**Fig. 1:**
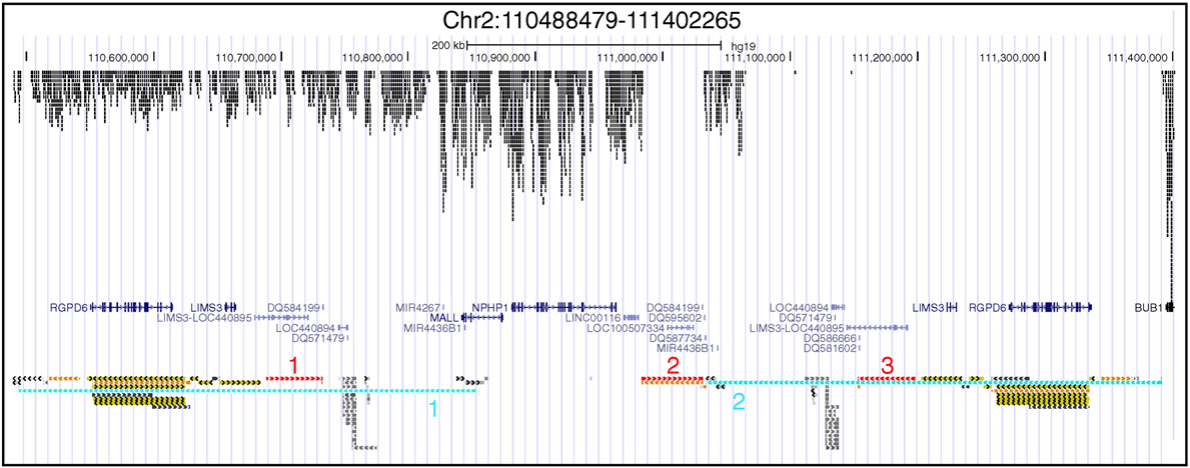
aCGH design at the human *NPHP1* locus. The aCGH design is shown as a UCSC Genome Browser custom track (top) together with the tracks of UCSC genes (middle) and Segmental Dups (bottom). The tracks were aligned according to genomic coordinates in the window Chr2:110488479-111402265. As illustrated, only the LCR copies on the left side of *NPHP1* were extensively covered with aCGH probes. In the bottom Segmental Dups track, multiple LCRs including the 45 kb LCRs and 358 kb LCRs are presented for this region. We focus on the three copies of the 45 kb LCRs (highlighted in red) and two copies of the 358 kb LCRs (highlighted in blue) [1] in the haploid reference genome. The different copies of each LCR group were annotated on the Segmental Dups track with red and blue numbers, respectively.

aCGH was designed in an 8X60K format using the Agilent SureDesign website (https://earray.chem.agilent.com/suredesign/, AMADID# 032837), based on human reference genome hg19. High-density aCGH probes were used to tile the human *NPHP1* locus and flanking regions. There are two major groups of LCRs flanking the gene *NPHP1*, each group consisting of LCR pairs that are > 99.6% identical [1]. Because of the high sequence similarity, we tiled aCGH probes from only one copy of the LCR sequences at the proximal side of *NPHP1* (Fig. 1). In this way, a clear visualization of copy number changes can be obtained by focusing on one of the two regions with high sequences similarity.

### 2.3 aCGH — experimental procedures

Thirty-two samples, as described above, were treated as “test samples” to compare with the universal control DNA sample of NA10851. For Agilent aCGH in 8x60K format, 900 ng of DNA was used for both test and control samples. Experimental steps of aCGH, including DNA fragmentation, DNA labeling and clean-up, array hybridization, and array washing and scanning were performed following the published protocol [2]. The image obtained from slide scanning was processed by Feature Extraction Software version 11.5 (Agilent Technologies) with default settings to generate feature extraction (FE) files. Agilent Genomic Workbench version 7.0 (Agilent Technologies) was used to process the FE files for copy number analysis. Copy number was determined by normalized log_2_ ratio (LR) of Cy5/Cy3 fluorescence signal of each probe. ADM-2 algorithm with threshold 4.0 was used to call CNVs.

### 2.4 aCGH — data analysis

The LR of each probe represents the relative DNA dosage level of Cy5 (test) versus Cy3 (control). For a DNA duplication, LR are calculated as log_2_(3/2), which equals ~ 0.58; while for a heterozygous DNA deletion, LR are calculated as log_2_(1/2), which equals − 1.0. Moreover, an arbitrary number (> 5) of consecutive probes are needed to support the call of a copy number gain or loss with high confidence. The CNVs called by the Agilent Genomic Workbench program were inspected manually. Mean LR value of consecutive probes in a designated region was calculated to estimate copy number.

**Fig. 2:**
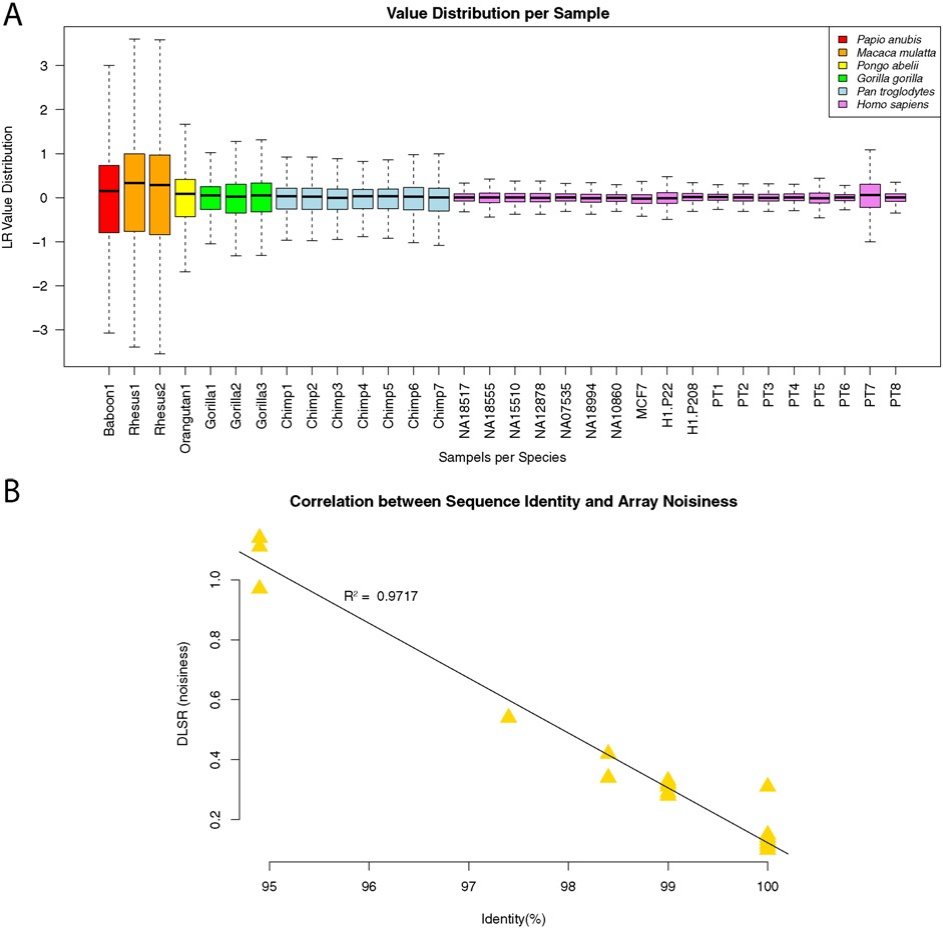
Assessment of aCGH data quality. A. Value distribution of log_2_ ratios (LR) shown in box plots. Thirty-two samples from six species were analyzed. The wide value distribution observed in PT7 may be due to low DNA quality. The color annotations for each species are shown on the top right of the figure. B. Correlation between sequence identity and aCGH data quality. DLRS (derivative log ratio spread) is a measurement of standard deviation of the differences between adjacent points (noisiness) in log ratio data. The DLRS (Y-axis) is plotted against sequences identity (X-axis). Each gold triangle represents a sample. A strong correlation (R^2^ = 0.9717) is observed.

All the test DNA samples of human and nonhuman primates were compared with DNA sample of NA10851, a human individual, by aCGH. The interspecies aCGH generated much higher experimental noise than intraspecies aCGH. The LR value distribution calculated for each sample illustrated a broader (noisier) LR value distribution in nonhuman primates than humans (Fig. 2A). The “noisiness” of data strongly correlated with the sequence identity between the two (test and control) samples being compared (Fig. 2B).

**Fig. 3:**
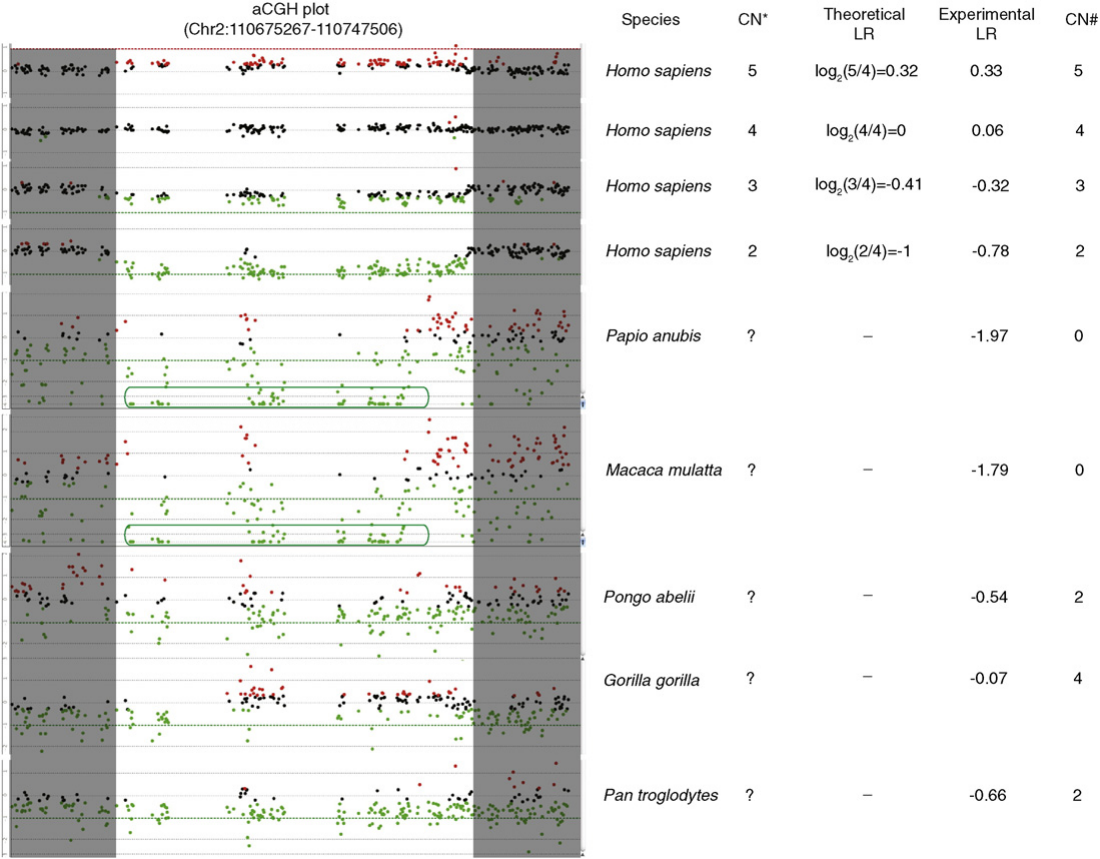
Examples of aCGH data for each species. Shown are aCGH LR plots of four human DNA samples with different copy number of the 45 kb LCRs and one nonhuman primate sample of each species. Red dots, probes with LR ≥ 0.25; black dots, probes with − 0.25 < LR < 0.25; green dots, probes with LR ≤ − 0.25. The human 45 kb LCRs is in the region flanked by the shaded areas. The open green boxes surrounding plots in the 5th and 6th aCGH panels include probes with LR value lower than − 2.0, indicating homozygous DNA losses. The species, copy number indicated by previous studies (CN*), theoretical LR calculated using CN*, experimental LR value based on the actual aCGH data from the current study, and the estimated copy number from the experimental LR value (CN#) are shown on the right side of the aCGH plots. The symbol “?” denotes unknown copy number before aCGH analysis.

Copy number analysis was focused on the human 45 kb LCRs and its nonhuman primate orthologues. Human individuals with known copy numbers of the 45 kb LCRs, ranging from 2 to 5, were used for “proof of principle” analysis. Results were consistent between the theoretical and experimental LR values, and consequently a reliable copy number estimation consistent with the copy number validated previously [3] in each individual was obtained using our method (Fig. 3 top four panels). The hybridization quality decreases as the sequences of the test and control samples become more divergent. As a result, the signals become more dispersed. In baboon and rhesus macaque, despite the noisy hybridization, the data fall far below the heterozygous deletion (− 1.0) level, and the mean LR calculated for the 45 kb LCRs region was also largely lower than − 1.0 (Fig. 3 5th and 6th panels). The evidence above suggests either a homozygous loss of this 45 kb LCR in both the baboon and rhesus macaque tested here, or that this LCR never evolved in these species. Hybridization for orangutan or chimpanzee versus human yielded data with better quality, represented by less “noisiness” probably due to less sequence divergence from human; and the mean LR calculations for the 45 kb LCRs region were − 0.54 and − 0.66, which were more consistent with a low copy number (*n* = 2) in these samples (Fig. 3 seventh and ninth panels). The hybridization for gorilla resulted in a mean LR of − 0.07, indicating an equal copy number for the 45 kb LCRs and its orthologues in the human and gorillas being tested (Fig. 3 eighth panel).

## 3 Discussion

aCGH can be designed with high-density probes interrogating the regions of interest such as exons. This kind of array has been widely implemented in clinical testing for small exonic and even mosaic CNV detection [4], [5]. Targeted aCGH has also been used in investigating regions with evolutionary significance, as exemplified in the association study of DUF1220-domain CNVs with human brain size [6]. Moreover, aCGH can be implemented with SNP array in a single assay to maximize its variant detection ability [7]. In our study, we used a customized high-density aCGH to investigate higher-order copy number changes of LCRs, which are not typically targeted by routine aCGH designs. This allows precise copy number estimation for LCRs. We also used this array to estimate copy number changes of the target LCRs during primate evolution. The interspecies aCGH data should be analyzed with great caution because of the hybridization noise due to divergent DNA sequences being compared. It is always recommended that aCGH calls be further validated by orthogonal experimental approaches including breakpoint PCR, fluorescence *in situ* hybridization or chromosome analysis. The copy number findings in our study were validated using orthogonal datasets from cDNA aCGH and whole genome sequencing [1], [8], [9]. Restricted by its intrinsic nature, aCGH does not readily detect copy number neutral events, such as inversions, balanced translocations and absence of heterozygosity.

Our dataset can be useful to scientists who are interested in studying genomic architectures and/or evolution histories of complex regions similar to the human *NPHP1* locus, a region that exhibits diverse structural variant haplotypes. Moreover, our data underscore the value of aCGH in studying interspecies copy number variations [8].

## Conflict of interest

JRL has stock ownership in 23andMe, Ion Torrent Systems, and Lasergen, Inc., is a paid consultant for Regeneron Pharmaceuticals and is a co-inventor on multiple United States and European patents related to molecular diagnostics for inherited neuropathies, eye diseases and bacterial genomic fingerprinting. The Department of Molecular and Human Genetics at Baylor College of Medicine derives revenue from the chromosomal microarray analysis (CMA) and clinical exome sequencing offered in the Medical Genetics Laboratory (MGL; http://www.bcm.edu/geneticlabs/). The MGL recently announced a joint venture with Miraca Holdings of Japan.
